# Comparison of the Accuracy of CBCT and MDCT Imaging Modalities in Determining the Distance between the Incus and the Facial Nerve and the Round Window and the Oval Window: A Cadaveric Cross-sectional Study -

**DOI:** 10.31661/gmj.vi.3919

**Published:** 2025-12-16

**Authors:** Sanaz Sharifishoshtari, Nader Saki, Zohreh Roozbahani, Yasamin Mehrab, Ali Hesari

**Affiliations:** ^1^ Department of Oral and Maxillofacial Radiology, Faculty of Dentistry, Ahvaz Jundishapur University of Medical Sciences, Ahvaz, Iran; ^2^ Hearing Research Center, Ahvaz Jundishapur University of Medical Sciences, Ahvaz, Iran

**Keywords:** MDCT, CBCT, Round Window, Oval Window, Facial Nerve, Incus, Cochlear Implant

## Abstract

**Background:**

Hearing loss is the most common birth defect, and cochlear implants are an effective treatment for severe sensorineural loss. This study compared CBCT and MDCT accuracy in measuring key ear structures to aid cochlear implant planning.

**Materials and Methods:**

In this cadaveric cross-sectional laboratory study, 12 temporal bones along with their covering soft tissue in the archive of the ENT department of Imam Khomeini Hospital in Ahvaz were studied. Imaging was performed using high-resolution CBCT, low-resolution CBCT, and MDCT modalities. Measurements were performed by an oral and maxillofacial radiologist and a general radiologist. The obtained data were analyzed descriptively and analytically in SPSS21 software.

**Results:**

The results revealed a high agreement between the data related to the distance of the incus from the facial nerve and the distance of the oval window from the round window in High-resolution CBCT, Low-resolution CBCT, and MDCT images. However, there was a significant difference in the measurement of MDCT and low-resolution CBCT compared to high-resolution CBCT. There was no significant difference in the measurement between MDCT and low-resolution CBCT.

**Conclusion:**

Based on our study and some previous studies on quantitative measurements of CBCT and MDCT imaging, low-resolution CBCT can be more reliably replaced by MDCT and achieve acceptable results compared to MDCT using a lower radiation dose. More studies are needed regarding the significant difference between high-resolution CBCT and MDCT, but the higher resolution of the borders in high-resolution CBCT images may be the reason for this result.

## Introduction

Hearing loss is the most common birth defect and one of the most common sensorineural defects in humans [1]. Because of its high prevalence, understanding its underlying causes and potential treatments is a crucial area of medical research. Several etiological factors, including non-syndrome genetic diseases, are implicated in various types of deafness. Over the past few decades, advancements in auditory science have significantly transformed approaches to hearing rehabilitation. The introduction of cochlear implants has been proven to be a safe, effective, and cost-effective method for treating severe to profound sensorineural hearing loss [2].

These devices have enabled individuals with profound deafness to regain auditory perception, thereby improving communication and quality of life. The round window (RW), connecting the end of the scala tympani (inside the inner ear) to the middle ear, functions as a window that equalizes the pressure in the inner ear and affects hearing with its vibrations [3]. Due to its anatomical role, the RW serves as a critical access point during cochlear implantation procedures. Nowadays, the preferred surgical method for cochlear implantation is through the round window, as it provides a safe and non-traumatic access route for electrode placement and has the highest potential for preserving residual hearing. However, anatomical variations in the RW region can make it challenging [4]. Therefore, precise preoperative imaging and anatomical assessment are essential to minimize surgical complications [4].

The oval window (OW) or fenestra vestibuli is a kidney-shaped opening located superiorly and posteriorly of the nose, leading from the tympanic cavity to the vestibule of the inner ear, and occupied by the base of the stapes. Its long diameter is horizontal and its convex margin is directed upward [5]. Together with the round window, the oval window plays an essential role in sound transmission and pressure regulation within the cochlea. The incus is a member of the ossicular chain in the mammalian middle ear that couples acoustic signals from the tympanic membrane to the inner ear [6]. This bone has a large trunk and two processes. Its trunk articulates anteriorly with the malleus and the distal end with the head of the stapes [7]. The coordinated motion of these ossicles ensures efficient mechanical transmission of sound waves to the cochlea. The facial nerve (VII) has a large motor root and a small sensory root that traverses the posterior cranial fossa and leaves the cranial fossa through the internal auditory canal. It also innervates the stapes muscle of the ear [8]. Because of its proximity to the middle and inner ear structures, the facial nerve is particularly vulnerable during otologic surgeries. The primary trunk of the facial nerve runs downward within the facial canal immediately after its junction with the posterior wall of the middle ear. This canal gives rise to a prominence on both the posterior and medial walls seen horizontally above the nose and oval window [7].

Understanding this complex anatomical relationship is vital for surgeons to avoid iatrogenic injury. Facial nerve palsy is a rare but devastating complication of CI surgery. Its rate varies from 0.67% to 1.2% [9]. Hence, detailed imaging and anatomical mapping have become integral to pre-surgical planning. Recent advances in CT scanning technology have enabled us to accurately measure the length and thickness of sections of ear structures individually. The information obtained from these images has minimized damage to the nerve and cochlea [10].

Multidetector computed tomography (MDCT) is a radiological evaluation method for diagnostic and therapeutic applications of hearing loss. Despite its diagnostic accuracy, concerns about radiation exposure have prompted the search for alternative imaging modalities. One of its major disadvantages is that it exposes patients to high doses of radiation. Cone beam computed tomography (CBCT) is increasingly used in dental surgery [11] and craniofacial surgery [12]. CBCT is associated with a lower radiation dose than MDCT [13]. Consequently, researchers have begun to explore its potential application in otologic imaging. Some studies have examined the feasibility of using CBCT in otologic imaging [14][15][16]. One of the advantages of CBCT in otologic imaging compared to MSCT is the possibility of limiting the acquisition of image data to only the region of interest (ROI), leading to lower patient radiation exposure per examination [17].

This advantage makes CBCT particularly appealing for pediatric and follow-up imaging, where cumulative radiation dose is a concern. Several comparative studies of MDCT and CBCT on human temporal bone specimens have been performed in vitro in the follow-up of middle ear prostheses, active middle ear implants, cochlear implants, and bone-borne hearing aids [18][19][20]. Reports suggest that the analysis of temporal bone structures using CBCT is satisfactory and associated with relatively low radiation doses (18). CBCT is a vital solution to reduce high radiation exposure when frequent radiographic examinations and patient follow-up are necessary [21]. However, further comparative studies are still required to establish its diagnostic equivalence and clinical safety in cochlear implant planning. No study has been conducted to compare the distance between the oval window and the incus and the distance between the incus and the facial nerve before cochlear implant surgery using MDCT and CBCT imaging. Thus, the present study was conducted to plan for cochlear implant treatment using CBCT and MDCT imaging methods to investigate the possibility of using CBCT with a lower radiation dose for safer and more accurate planning of ear implant treatment.

## Materials and Methods

The present cadaveric study was conducted with an applied purpose and designed as a cross-sectional study. The study population consisted of 12 human temporal bones, including their covering soft tissues, obtained from the anatomical archive of the Dissection Room in the Department of Otolaryngology (ENT) at Imam Khomeini Hospital, Ahvaz, Iran. The sample size was determined based on prior research findings by Dehmani Kusa et al. (2022) and the recommendations of a statistical consultant, using MedCalc statistical software with an alpha error of 5% and a statistical power of 80%.

The inclusion criteria consisted of temporal bones with intact helical structures. This study was conducted on cadaveric temporal bones separated from the skulls, and therefore did not involve living human participants or animal subjects; ethical approval was not required, consistent with institutional and national research guidelines.

The imaging modalities utilized in this study included multidetector computed tomography (MDCT) and cone-beam computed tomography (CBCT). For each imaging procedure, the temporal bones were stabilized within plastic containers. The MDCT imaging was performed using a Siemens Sensation 64-slice helical CT scanner, with parameters set at 120 kVp, 70 mAs, 0.6-mm slice thickness, and a pitch of 1.4.

For CBCT imaging, a NewTom VGi (Quantitative Radiology, Verona, Italy) system was employed. The containers containing the temporal bones were securely fixed within the CBCT unit using adhesive tape. Images were acquired using a field of view (FOV) of 8 × 12 cm, with exposure parameters of 110 kVp, and acquisition times of 3.6 seconds for low-resolution scans and 5.4 seconds for high-resolution scans. All images were saved and processed using NNT software (NewTom Imaging Suite).

**Figure-1:**
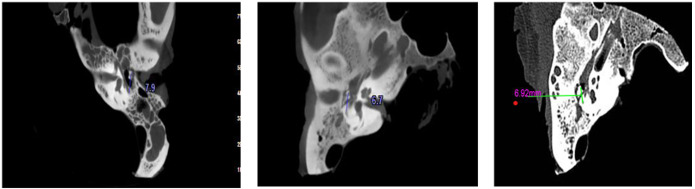
Figure 1. MDCT image (right), Low-resolution CBCT (middle), and High-resolution CBCT (left) for measuring the distance of the incus from the facial nerve

CBCT images were evaluated by a board-certified oral and maxillofacial radiologist, while MDCT images were assessed by a general radiologist, both using a 14-inch ASUS LED monitor (1920 × 1080 resolution) in a semi-darkened, controlled viewing environment. For each image, the distances between the round window and the oval window, and between the incus and the facial nerve, were measured using the digital ruler tool available within the respective CT and CBCT software platforms (see Figure-1 for illustration).

The obtained data were analyzed descriptively and analytically in SPSS software (Chicago, SPSS INC, SPSS V23 for Windows, USA, IL). Shapiro-Wilk statistical tests, Pearson correlation coefficient, and paired t-test were used to examine the data and their normality. Descriptive statistics including mean, standard deviation, table and graph, and inferential statistics including analysis of variance (ANOVA) test or its non-parametric equivalent were performed using SPSS-21 software. A significance level of less than 5% was considered.

## Results

**Table:** Table1. Descriptive Statistics for Measured Distances (mm) in MDCT, High-resolution CBCT, and Low-resolution CBCT Imaging

**Measurement**	**Imaging Modality**	**Mean**	**SD**	**Median**
	MDCT	4.296	0.402	4.355
Incus-Facial Nerve	High-resolution CBCT	4.300	0.388	4.400
	Low-resolution CBCT	4.617	0.413	4.700
	MDCT	7.017	0.383	6.900
Oval-Round Window	High-resolution CBCT	7.300	0.354	7.250
	Low-resolution CBCT	7.043	0.391	6.975

**Table:** Table2. Pearson Correlation Coefficients Between Imaging Modalities for Measured Distances

**Measurement**	**Comparison**	**r**	**P-value**
	MDCT × High-res CBCT	0.783	0.003
Incus-Facial Nerve	MDCT × Low-res CBCT	0.906	< 0.001
	High-res × Low-res CBCT	0.918	0.003
	MDCT × High-res CBCT	0.884	< 0.001
Oval-Round Window	MDCT × Low-res CBCT	0.784	< 0.001
	High-res × Low-res CBCT	0.884	< 0.001

**Table:** Table3. Paired t-Test Results Comparing Measurement Accuracy Between Imaging Modalities

**Measurement**	**Comparison**	**MD**	**SD**	**95% CI (Lower-Upper)**	**t**	**df**	**P-value**
	High-res CBCT vs MDCT	0.321	0.269	0.150-0.492	4.135	11	0.002
Incus-Facial Nerve	Low-res CBCT vs MDCT	0.004	0.172	-0.105-0.113	0.084	11	0.935
	High-res vs Low-res CBCT	-0.317	0.164	-0.421--0.212	-6.680	11	< 0.001
	High-res CBCT vs MDCT	0.257	0.183	0.141-0.374	4.885	11	< 0.001
Oval-Round Window	Low-res CBCT vs MDCT	-0.026	0.255	-0.188-0.136	-0.352	11	0.732
	High-res vs Low-res CBCT	-0.283	0.233	-0.431--0.135	-4.214	11	0.001

MD, mean difference; SD, standard deviation. High-res, High-resolution; Low-res, Low-resolution

The mean distance of the incus from the facial nerve was 4.296 ± 0.402 mm in MDCT images, 4.300 ± 0.388 mm in high-resolution CBCT images, and 4.617 ± 0.413 mm in low-resolution CBCT images. Similarly, the mean distance between the oval and round windows was 7.017 ± 0.383 mm in MDCT, 7.300 ± 0.354 mm in high-resolution CBCT, and 7.043 ± 0.391 mm in low-resolution CBCT (Table-1).

### Correlation Analysis

Pearson correlation analyses demonstrated a strong, statistically significant correlation between the measurement modalities for both variables. For the incus-facial nerve distance, correlations were significant between MDCT and high-resolution CBCT (r=0.783, P=.003), MDCT and low-resolution CBCT (r=0.906, P<.001), and high-resolution and low-resolution CBCT (r=0.918, P=.003). For the oval-round window distance, correlations were also significant between MDCT and high-resolution CBCT (r=0.884, P<.001), MDCT and low-resolution CBCT (r=0.784, P<.001), and high-resolution and low-resolution CBCT (r=0.884, P<.001) (Table-2).

Paired-sample t-tests were used to compare the accuracy of measurements among imaging modalities (Table-3). For the incus-facial nerve distance, a significant difference was observed between high-resolution CBCT and MDCT (P=.002), and between high-resolution and low-resolution CBCT (P<.001). However, the difference between low-resolution CBCT and MDCT was not significant (P=.935). For the oval-round window distance, a significant difference was found between high-resolution CBCT and MDCT (P<.001), as well as between high-resolution and low-resolution CBCT (P=.001). The difference between low-resolution CBCT and MDCT was not significant (P=.732).

## Discussion

The present cadaveric study demonstrated that both MDCT and CBCT provided reliable and consistent measurements of key temporal bone anatomical distances, specifically between the incus and the facial nerve and between the oval and round windows. The high-resolution CBCT (HR-CBCT) images produced measurements with the closest agreement to MDCT, supported by strong positive correlations and statistically significant paired comparisons. These findings emphasize the diagnostic value of HR-CBCT in visualizing delicate middle ear structures essential for surgical planning, particularly in otologic and cochlear implantation procedures. This result aligns closely with Sharifishoshtari et al. (2024), who found comparable accuracy between HR-CBCT and MDCT when measuring the linear distance between the stapes and round window, as well as the incudostapedial joint thickness [23].

Both studies demonstrate that HR-CBCT can serve as a viable alternative to MDCT, offering high spatial resolution while reducing radiation exposure and cost. However, in contrast to Sharifishoshtari et al.’s findings, which reported slightly higher HR-CBCT values for certain distances, our data showed minimal measurement discrepancies between the two modalities, possibly due to differences in anatomical landmarks and measurement calibration protocols. Further comparison with anatomical investigations, such as the work of Jain et al. (2018) [24], reinforces the clinical relevance of our findings. Jain and colleagues [24] emphasized that the anatomical relationship between the round window, oval window, and facial nerve plays a decisive role in determining the complexity of cochlear implant insertion. Our study supports this anatomical observation by providing quantitative imaging evidence that the mean oval-round window distance (approximately 7 mm) falls within the normal range reported in dissection-based studies. This correspondence validates the imaging-based measurements of CBCT and MDCT against direct anatomical references. Moreover, Jain et al. (2017) demonstrated that variations in the facial recess and round window visibility significantly influence electrode insertion safety and accessibility [25]. The narrow differences in measurements observed in our HR-CBCT and MDCT data, compared to the broader anatomical variability documented in cadaveric dissections, suggest that CBCT imaging can reliably capture these subtle morphological variations without invasive intervention. Such precision is particularly beneficial for preoperative evaluations where direct access to the structures is limited.

From a broader perspective, the present results contribute to the evolving understanding of CBCT’s role in otologic imaging and its application in cochlear implantation planning. Studies such as Bernardo et al. (2013) [26] and Zou et al. (2014, 2015) [27][28] have highlighted the necessity of accurately visualizing the facial nerve and cochlear fine structures through various surgical and imaging approaches. Bernardo et al. [26] showed that an optimal surgical route depends heavily on the degree of exposure and anatomical orientation of the facial nerve, findings that harmonize with our observation that HR-CBCT yields high-definition visualization of the facial recess and adjacent neural structures. Similarly, Zou and colleagues verified, using both experimental CBCT and high-resolution microtomography (μCT), that CBCT reliably delineates the scala tympani, osseous spiral lamina, and round window niche. The consistency between their microstructural imaging results and our quantitative data further validates CBCT’s anatomical accuracy. Together, these findings establish that HR-CBCT, while maintaining a lower radiation dose and greater accessibility, provides measurement reliability and spatial resolution comparable to MDCT and even μCT in certain parameters. Consequently, HR-CBCT can be recommended as an effective, minimally invasive imaging tool for cochlear implantation planning and other middle ear assessments where fine anatomical precision is crucial.

## Conclusion

The present study compared the accuracy of CBCT and MDCT images in measuring the distance between the incus and the facial nerve and the distance between the round window and the oval window of the ear. The results revealed a significant relationship between the results obtained in measuring the distance between the incus and the facial nerve and the distance between the round window and the oval window of the ear between CBCT and MDCT. The results of both imaging modalities showed high agreement. CBCT can be used as a safe and optimal imaging tool for ear and inner ear measurements due to the lower radiation dose, higher resolution, and acceptable image quality.

## Conflict of Interest

None.
